# Interleukin-37 protects against acinar cell pyroptosis in acute pancreatitis

**DOI:** 10.1172/jci.insight.161244

**Published:** 2022-11-08

**Authors:** Nan Ma, Chenchen Yuan, Juanjuan Shi, Qingtian Zhu, Yang Liu, Xiaojie Ma, Baiqiang Li, Weijuan Gong, Jing Xue, Guotao Lu, Weiqin Li, Jieshou Li

**Affiliations:** 1 Department of Critical Care Medicine, Research Institute of General Surgery, Affiliated Jinling Hospital, Medical School of Nanjing University, Nanjing, China.; 2 Pancreatic Center, Department of Gastroenterology, and; 3 Yangzhou Key Laboratory of Pancreatic Disease, Institute of Digestive Diseases, The Affiliated Hospital of Yangzhou University, Yangzhou University, Yangzhou, China.; 4 State Key Laboratory of Oncogenes and Related Genes, Stem Cell Research Centre, Ren Ji Hospital, School of Medicine, Shanghai Jiao Tong University, Shanghai, China.; 5 Department of Critical Care Medicine, Jinling Hospital, Medical School of Southeast University, Nanjing, China.

**Keywords:** Gastroenterology, Inflammation, Cytokines, Pharmacology

## Abstract

Acute pancreatitis (AP) is a local and/or systemic inflammatory disease that starts with acinar cell injury and necrosis; it has no effective medical treatment and thus remains a life-threatening condition. Interleukin-37 (IL-37), a natural immunomodulator, has demonstrated an antiinflammatory effect; however, the role of IL-37 in AP remains unknown. The serum IL-37 levels of 39 healthy controls and 94 patients with AP were measured. Cholecystokinin was applied to induce pancreatic acinar cell injury in vitro. Classical experimental AP models, such as caerulein, l-arginine, and taurolithocholic acid 3-sulfate disodium salt, were included in the in vivo study. A transgenic mouse model with the IL-37 gene and administration of recombinant IL-37 were used to further investigate the function of IL-37 in AP. Pancreas-specific gasdermin D–knockout (GSDMD-knockout) mice were used to explore the protective mechanism of IL-37. Our results showed that serum IL-37 levels in humans were negatively correlated with the severity of AP. Furthermore, IL-37–transgenic mice and supplementation with recombinant IL-37 could both protect against AP. Mechanistically, IL-37 was able to suppress pyroptosis of injured acinar cells, and specific depletion of GSDMD in the pancreas counteracted the protective effect of IL-37. Our study demonstrates that IL-37 protects against acinar cell pyroptosis in AP.

## Graphical abstract

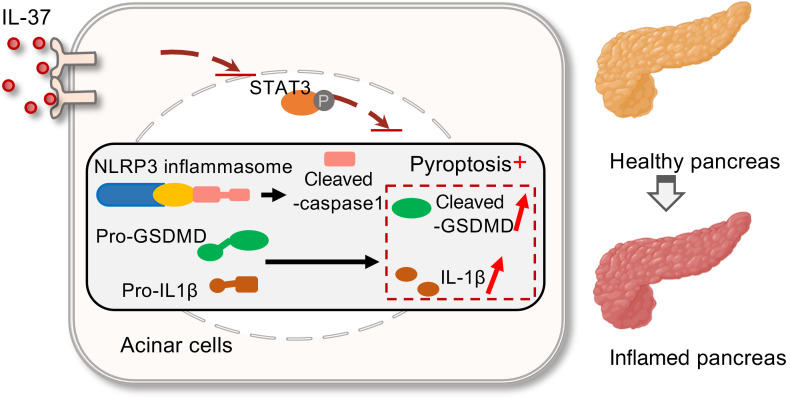

## Introduction

Acute pancreatitis (AP) is an urgent and severe inflammatory disease that is regarded as the most common gastrointestinal emergency (1). Recent studies have demonstrated that AP shows an accelerating incidence accompanied by higher mortality (2, 3). High mortality is primarily attributed to pancreatic necrosis and organ failure (4). Despite efforts to design therapeutic strategies and drug development, the current clinical treatment of AP patients is still limited to traditional support and surgery (5, 6).

The knowledge of the pathology of AP is ever changing. Pathologically, AP is characterized by acinar cell death and a cascade inflammatory response (7). Our recent research has uncovered the crucial role of acinar cell pyroptosis in the pathogenesis of AP and defined pyroptosis as a promising therapeutic target by establishing pancreas-specific pyroptosis execution protein gasdermin D (GSDMD) in knockout mice (8). Pyroptosis is a form of proinflammatory programmed cell death (9). However, little pharmaceutical research on AP has been conducted with the goal of targeting pyroptosis as a treatment strategy for AP (10). Therefore, therapy targeted at the pyroptosis execution protein GSDMD has yet to be studied in full.

Interleukin-37 (IL-37) is a newly described member of the interleukin family (11). Physiologically, IL-37 is widely expressed in blood, bone marrow, and pancreas of humans (12, 13). Serum IL-37 levels were elevated in patients with atherosclerosis (14) yet decreased in severe keloids (15) and severe COVID-19 (16). Moreover, serum IL-37 levels were elevated in nonseptic ICU patients compared with healthy controls, while decreased in septic patients (17). Presently, the function of IL-37 is not fully understood. IL-37 plays a definite antiinflammatory role in multiple inflammatory and metabolic disorders, such as diabetes (18), atherosclerosis (19), and inflammatory bowel disease (20, 21). A study on murine pneumococcal pneumonia, in contrast, demonstrated the proinflammatory role of IL-37 in the disease pathology (22). Ultimately, IL-37 elicits a broad antiinflammatory response, but its possible adverse effects in some disease pathologies need to be explored.

Here, we sought to explore the role of IL-37 in AP. We measured the level of serum IL-37 in patients with AP and analyzed its relationship with clinical characteristics. However, it is challenging to obtain human pancreatic tissue during an AP episode, so nearly all the early cellular events have been investigated using animal models (7, 23). Currently, mice are the most widely used species because of low-cost and available mouse strains with genetic engineering (8, 23). Since the mouse lacks a homologous gene for human IL-37, we herein investigated biological functions of IL-37 in experimental AP by using a mouse strain transgenic for human IL-37 (IL37tg) and recombinant human IL-37. The transgenic mouse strain was generated expressing human IL-37 in most cells driven by a cytomegalovirus (CMV) promoter (20, 24).

## Results

### Evaluation of serum IL-37 levels in healthy participants and patients with AP.

**Figure 1:**
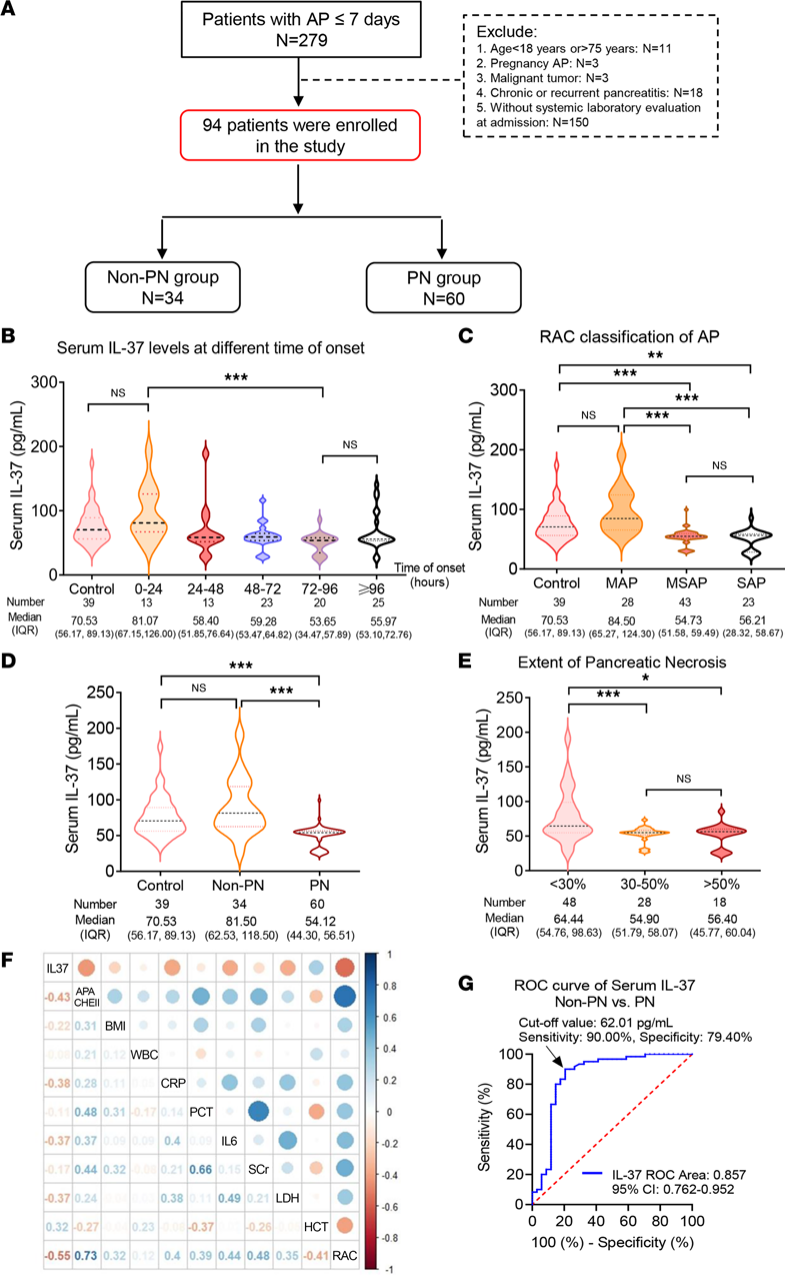
Serum IL-37 levels in AP patients. (**A**) Flowchart of the patients with AP in the clinical study. (**B**) Serum IL-37 levels in controls and AP patients at different times of onset. (**C**) ELISA analysis of serum IL-37 levels in healthy controls and different classification of AP patients (*n* = 94). (**D**) ELISA levels of control, non-PN, and PN groups. (**E**) Serum IL-37 levels in AP patients with different extents of pancreatic necrosis (PN). (**F**) Heatmap of correlation coefficients between serum IL-37 levels and other clinical indices at admission. (**G**) Receiver operating characteristic (ROC) curve analysis of serum IL-37 levels for diagnosing PN. Data were analyzed using Kruskal-Wallis test. Data are presented as median (IQR), and statistical significance is denoted as *P < 0.05, **P < 0.01, and ***P < 0.001. APACHE II, Acute Physiology and Chronic Health Evaluation II; CRP, C-reactive protein; HCT, hematocrit; LDH, lactate dehydrogenase; MAP, mild AP; MSAP, moderately severe AP; PCT, procalcitonin; RAC, Revised Atlanta Classification; SAP, severe AP; SCr, serum creatinine; WBC, white blood cells.

A total of 94 patients diagnosed with AP were divided into a non–pancreatic necrosis (non-PN) group (*n* = 34) and a PN group (*n* = 60) (Figure 1A). Serum IL-37 levels were significantly decreased at 72–96 hours compared with those at 0–24 hours (*P* < 0.001) and then started to rise after 96 hours from onset; however, there was no significant difference between the ≥96 hours and 72–96 hours groups (Figure 1B). AP was diagnosed according to the International Atlanta Symposium on Acute Pancreatitis (25). According to the occurrence of local or systemic complications, such as PN and organ failure, AP was further categorized into mild AP (MAP), moderately severe AP (MSAP), and severe AP (SAP). As shown in Figure 1C, serum IL-37 levels in healthy controls were detectable, and there was no significant change in MAP. However, serum IL-37 levels in the MSAP and SAP groups were significantly lower than those in controls and MAP. We further analyzed changes of serum IL-37 levels and other indices in the PN and non-PN groups. Serum IL-37 levels in controls were not significantly different from those in non-PN patients; however, serum IL-37 levels of control and non-PN patients were significantly higher than those of the PN group (Figure 1D). In addition, BMI (*P* = 0.044), serum lactate dehydrogenase (LDH) (*P* = 0.002), C-reactive protein (CRP) (*P* < 0.001), and IL-6 (*P* < 0.001) were significantly increased in the PN group (Supplemental Table 1; supplemental material available online with this article; https://doi.org/10.1172/jci.insight.161244DS1). The extent of PN is based on a radiological scoring system (26, 27). The extent of PN is also called the area of PN and is generally described by the PN score, which is <30% for mild, 30%–50% for moderate, and >50% for severe necrosis. We found that the extent of PN increased gradually accompanied by a decrease in serum IL-37 levels (Figure 1E).

A logistic regression model was established to evaluate the risk factors for PN. Definite risk factors associated with PN, such as several baseline variables (sex, age, BMI), clinical severity scores, and history of smoking or drinking, and definite laboratory markers such as white blood cells, CRP, IL-6, and serum creatinine (SCr), were involved in univariate analysis (Supplemental Table 1). Serum IL-37 levels did not significantly correlate with Acute Physiology and Chronic Health Evaluation (APACHE) II score and other laboratory indices (white blood cells, CRP, IL-6, SCr, LDH, and hematocrit) (Figure 1F). Multivariable analysis was established (Supplemental Table 1), suggesting that decreased serum IL-37 (*P* = 0.041) and an increase in APACHE II (*P* = 0.048) were significantly associated with occurrence of PN.

A receiver operating characteristic curve was used to analyze the prognostic efficiency of serum IL-37 for PN. As shown in Figure 1G, the area under the curve was 0.857, the 95% CI was 0.762–0.952, and the optimal cutoff value for IL-37 was 62.01 pg/mL (sensitivity 90.00%, specificity 79.40%). Consistent with changes of serum IL-37 in community-acquired pneumonia patients (28), reduced serum IL-37 levels might be a promising clinical biomarker in the early phase of disease. In conclusion, serum IL-37 levels have promising clinical predictive value for PN development.

### Reduced severity of experimental AP in IL37tg mice.

**Figure 2:**
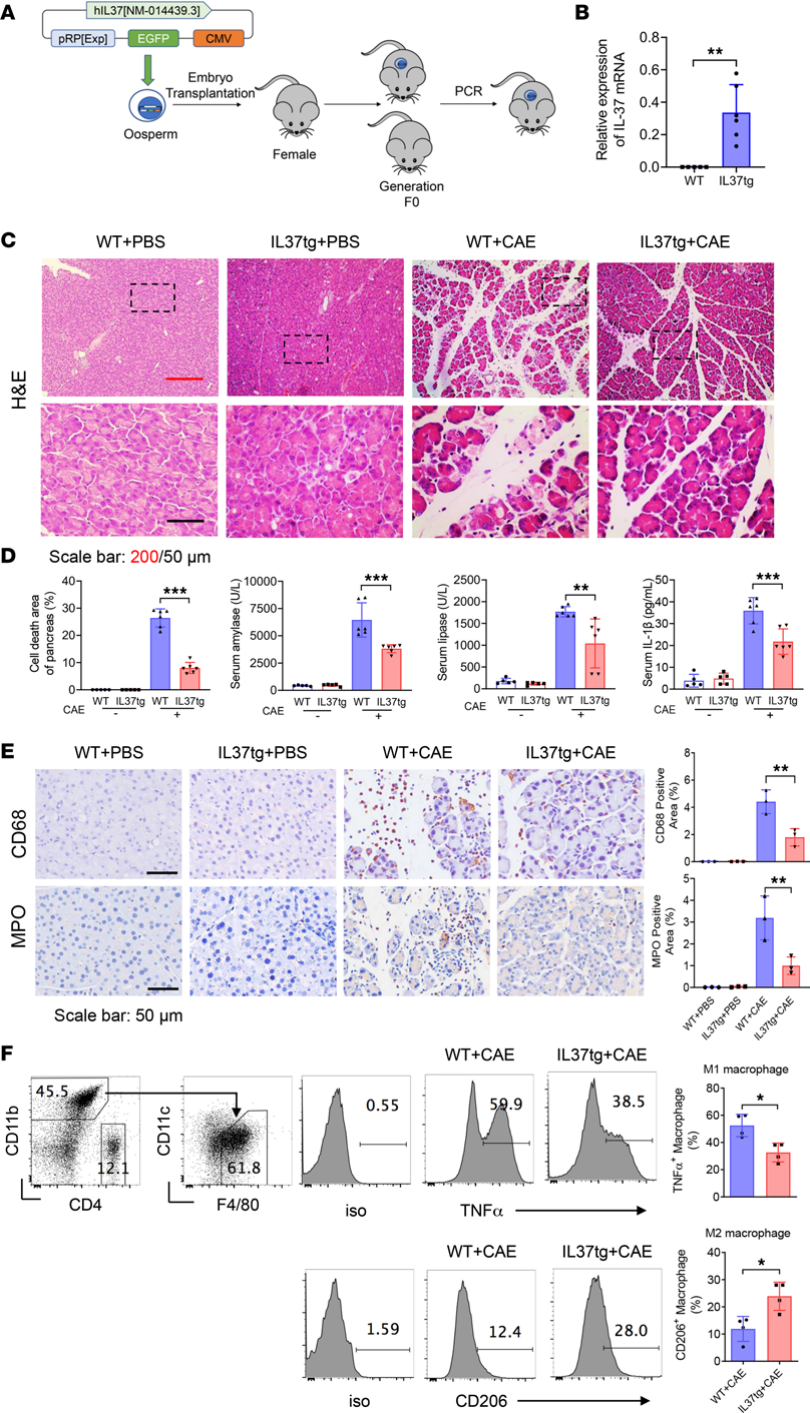
Reduced severity of experimental AP in IL37tg mice. (**A**) Schematic diagram of the generation of human IL-37 transgenic mice (IL37tg). (**B**–**F**) WT and IL37tg mice were injected with PBS and caerulein (CAE; 200 μg/kg, 1-hour intervals, 10 times total) to generate control and AP groups, respectively. Mice were harvested at 24 hours after the first injection. (**B**) Quantitative real-time PCR analysis of IL-37 mRNA expression in pancreatic tissues from IL37tg and WT littermates in normal condition. (**C**) H&E staining of pancreatic tissues from the indicated groups (*n* = 5–6 per group). Scale bars: 200 or 50 μm. (**D**) Percentages of pancreatic cell death area, and serum amylase, lipase, and IL-1β levels at 12 hours. (**E**) IHC staining of CD68 and MPO in pancreatic tissues, which marked macrophages and neutrophils, respectively (*n* = 3 per group). Scale bars: 50 μm. (**F**) Flow cytometry analysis of pancreatic leukocytes from the WT-CAE and IL37tg-CAE groups. Representative flow cytometry gating and the proportion of M1 (CD11b^+^F4/80^+^TNF-α^+^ of CD45.2^+^) and M2 (CD11b^+^F4/80^+^CD206^+^ of CD45.2^+^) macrophages (*n* = 4 per group). Experiments were repeated 3 times. Statistical comparisons were made using 1-way ANOVA or Student’s 2-tailed *t* test. Data are presented as mean ± SD, and statistical significance is denoted as *P < 0.05, **P < 0.01, and ***P < 0.001.

To explore the physiological and pathological effect of IL-37 in AP, we engineered transgenic mice expressing IL-37 (IL37tg) (Figure 2A). First, IL37tg mice were used to explore the effect of IL-37 on AP. IL37tg and WT mice were randomly divided into control and caerulein-induced (CAE-induced) AP groups. The mRNA levels of IL-37 were analyzed to verify the successful construction of the human IL-37–transgenic model (Figure 2B). Compared with that in the CAE-treated WT group, CAE-induced AP in the IL37tg group was significantly mitigated, which was illustrated by the reduction in pancreatic tissue necrosis in the transgenic mice (Figure 2, C and D, and Supplemental Figures 2 and 3). In addition, serum amylase, lipase, and IL-1β levels in the IL37tg-AP group were significantly reduced at 12 hours (Figure 2D). To observe inflammatory infiltration, we stained macrophages and neutrophils with anti-CD68 and anti-myeloperoxidase (anti-MPO) antibodies, respectively. The results showed that the infiltrated macrophages and neutrophils in the IL37tg-AP group were significantly fewer than those in the WT-AP group (Figure 2E). Moreover, pancreatic leukocytes were isolated and analyzed by flow cytometry. Compared with those in the WT-AP group, proinflammatory M1 (TNF-α^+^) macrophages were significantly reduced, while the proportion of antiinflammatory M2 (CD206^+^) macrophages was significantly increased, in the IL37tg-AP group (Figure 2F).

**Figure 3:**
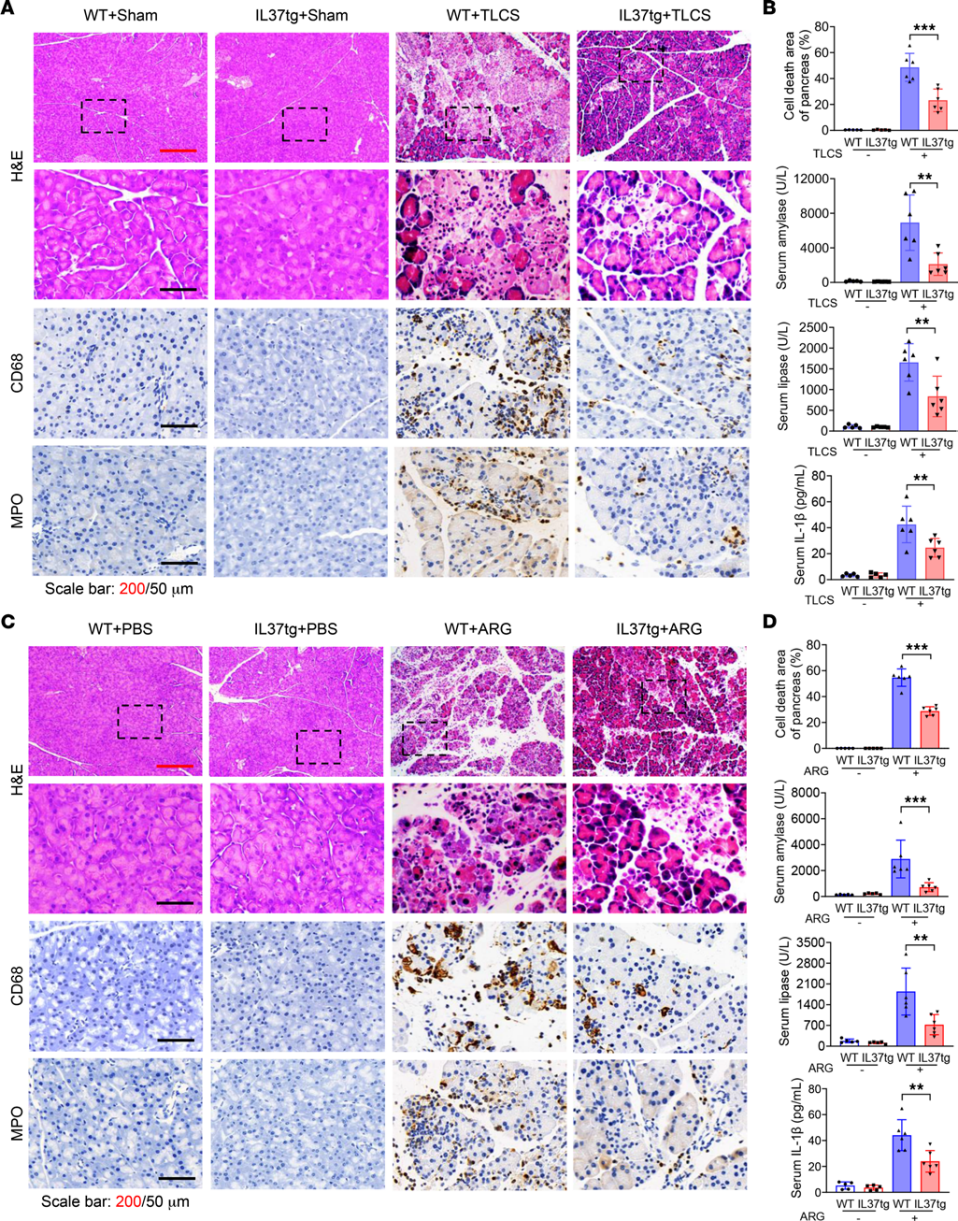
Protective effects of IL-37 in TLCS- and ARG-induced AP models. (**A** and **B**) Taurolithocholic acid 3-sulfate disodium salt (TLCS; 2.5%, 2 mg/kg) was injected in retrograde into the pancreatic bile duct to induce AP in IL37tg and WT mice. The sham group was used as the control in TLCS-induced experimental AP models. (**A**) H&E staining of pancreatic tissues from the indicated groups at 24 hours, and representative IHC staining of CD68 and MPO in pancreatic tissues. Scale bars: 200 or 50 μm. (**B**) Percentages of pancreatic cell death area, and serum amylase, lipase, and IL-1β levels at 24 hours (*n* = 5–6 per group). (**C** and **D**) WT and IL37tg littermates were given i.p. injections of 8% l-arginine (ARG; 3.3 g/kg, hourly, 3 times total) to induce AP, while an equivalent volume of PBS was injected into mice from the control group. (**C**) H&E staining at 72 hours, and representative IHC staining of CD68 and MPO in pancreatic tissues. Scale bars: 200 or 50 μm. (**D**) Percentages of pancreatic cell death area, and serum amylase, lipase, and IL-1β levels (*n* = 5–6 per group). Statistical comparisons were made using 1-way ANOVA. Data are presented as mean ± SD, and statistical significance is denoted as **P < 0.01, and ***P < 0.001.

Next, we investigated whether IL-37 was able to protect against severe AP (SAP). To this end, we introduced 2 SAP models by using taurolithocholic acid 3-sulfate disodium salt (TLCS) and l-arginine (ARG). Pathological injuries such as PN and inflammatory infiltrates in the IL37tg-SAP group were significantly alleviated compared with those in the WT-SAP group (Figure 3, A and C, and Supplemental Figure 4). Additionally, serum amylase and lipase levels were significantly lower in the IL37tg group than those of both the TLCS and ARG SAP models (Figure 3, B and D). Collectively, the efficacy of IL-37 was verified in all 3 in vivo classical AP models, which demonstrates the promising therapeutic role of IL-37 in AP.

Previous studies have reported that the mouse lacks a homologous gene for human IL-37 (29). We used an IL-37 human monoclonal antibody for immunohistochemical labeling on mice (WT and human-IL37tg) treated or not treated with CAE as previously (30) to assess the expression of IL-37 in pancreatic tissues of transgenic mice. IL-37 was definitely undetectable in pancreata of WT mice (Supplemental Figure 1A), but it was detectable in transgenic mice. IL-37 was significantly increased in experimental AP, and was expressed in acinar cells as well as locally invading immune cells.

Multiple studies have reported that IL-37 mainly derives from bone marrow (BM) (20, 31, 32). Since IL-37 transgene was randomly inserted into transgenic mice created using a CMV promoter, IL37tg mice showed no tissue- or cell-specific expression. To assess the contribution of BM-derived IL-37, we generated BM chimeric mice by engrafting WT recipients with either WT or IL37tg BM cells (WT→WT or IL37tg→WT mice, respectively). After 8 weeks of recovery, mice were treated with CAE to induce AP (Supplemental Figure 1B). The IL37tg→WT mice had alleviated experimental PN and reduced levels of serum amylase compared with the WT→WT group (Supplemental Figure 1, C and D). Overall, hematopoietic cell–derived IL-37 protected against AP.

### Supplementation with recombinant IL-37 protects mice from experimental AP.

**Figure 4:**
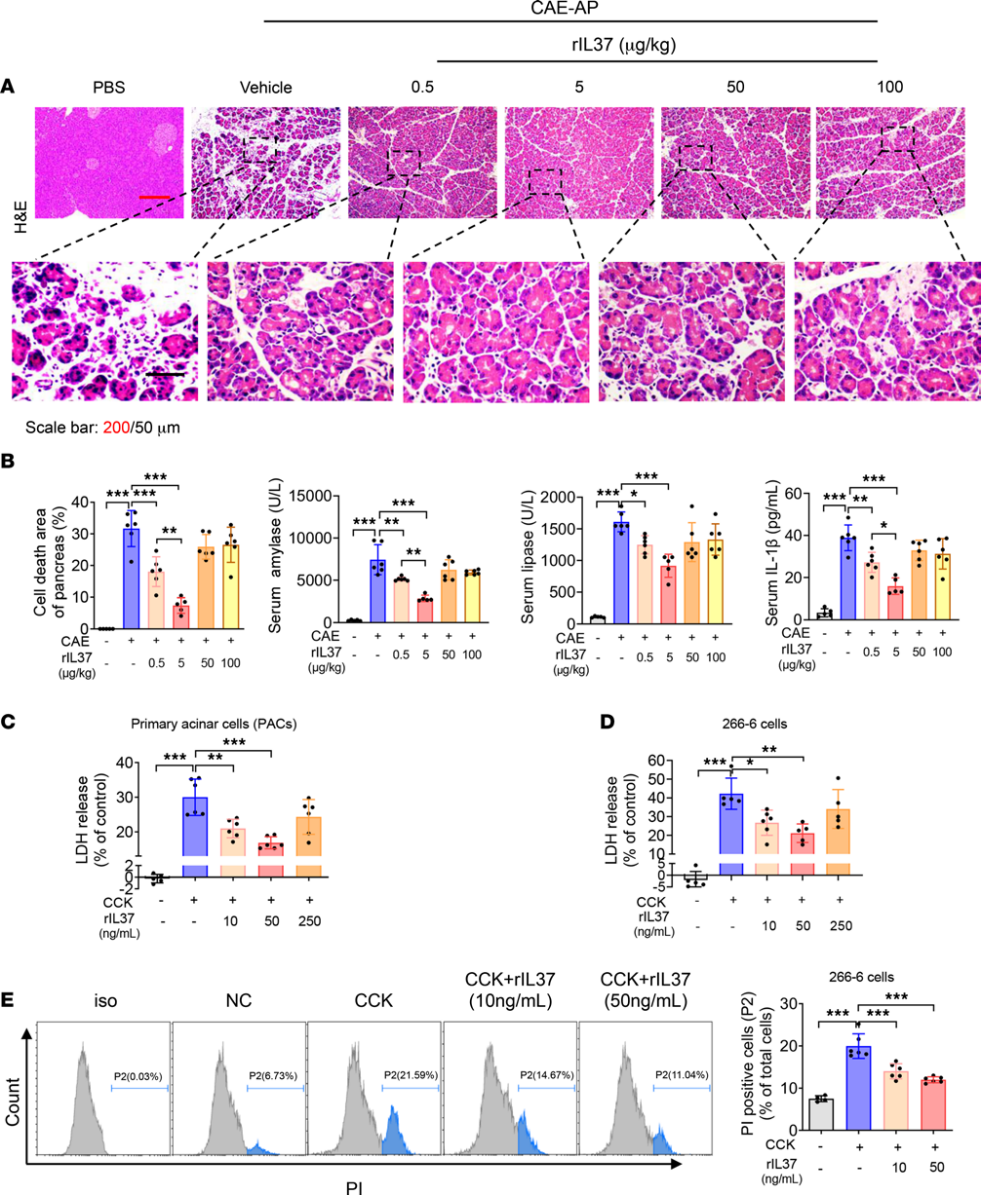
Recombinant IL-37 protects mice from experimental AP. (**A** and **B**) C57BL/6J WT mice were injected with CAE to induce AP, and after 1 hour, the indicated doses of rIL37 were administered (*n* = 5–6 per group). Mice injected with PBS were used as normal controls. All mice were sacrificed and harvested after 24 hours. (**A**) H&E staining of pancreatic tissue from the indicated mice. Scale bars: 200 or 50 μm. (**B**) Percentages of pancreatic cell death area, and serum amylase, lipase, and IL-1β levels at 12 hours. (**C** and **D**) Pancreatic acinar cell line 266-6 or primary acinar cells were treated with CCK, together with gradient doses of rIL37 for 12 or 6 hours, respectively. The levels of LDH release are shown. (**E**) 266-6 cells were treated with CCK together with rIL37 for 12 hours; then cells were harvested for propidium iodide (PI) staining. Representative flow cytometry histograms and the proportion of PI-positive cells are shown. Experiments were repeated 3 times. Statistical comparisons were made using 1-way ANOVA. Data are presented as mean ± SD, and statistical significance is denoted as *P < 0.05, **P < 0.01, and ***P < 0.001. NC, normal control.

We further investigated the effect of supplementation with recombinant IL-37 (rIL37) on AP. The groups were as follows: control group, AP group, and treatment groups of rIL37 protective dose gradients (0.5, 5, 50, and 100 μg/kg, 1 hour after the first CAE injection). The lower dose groups (0.5 and 5 μg/kg) showed a protective effect against pathological injury (Figure 4, A and B). Then we evaluated rIL37 at 1, 3, and 6 hours after CAE injection and observed significant protective effects at all these time points (Supplemental Figure 5). In addition, we treated mice with rIL37 at 1 hour before CAE injection and found that IL-37 had a preventive effect on experimental AP as well (Supplemental Figure 6).

Pancreatic acinar cells account for 95% of pancreatic tissues, and acinar cell injury has been considered an initial event of AP (33). We further verified whether rIL37 had direct effects on acinar cells. We found that rIL37 suppressed cholecystokinin-induced (CCK-induced) cell death in both acinar cell line 266-6 and primary acinar cells in vitro (Figure 4, C–E). In summary, IL-37 protected mice from experimental AP by alleviating pancreatic acinar cell death.

### IL-37 protects against AP by inhibiting GSDMD-mediated acinar cell pyroptosis.

**Figure 5:**
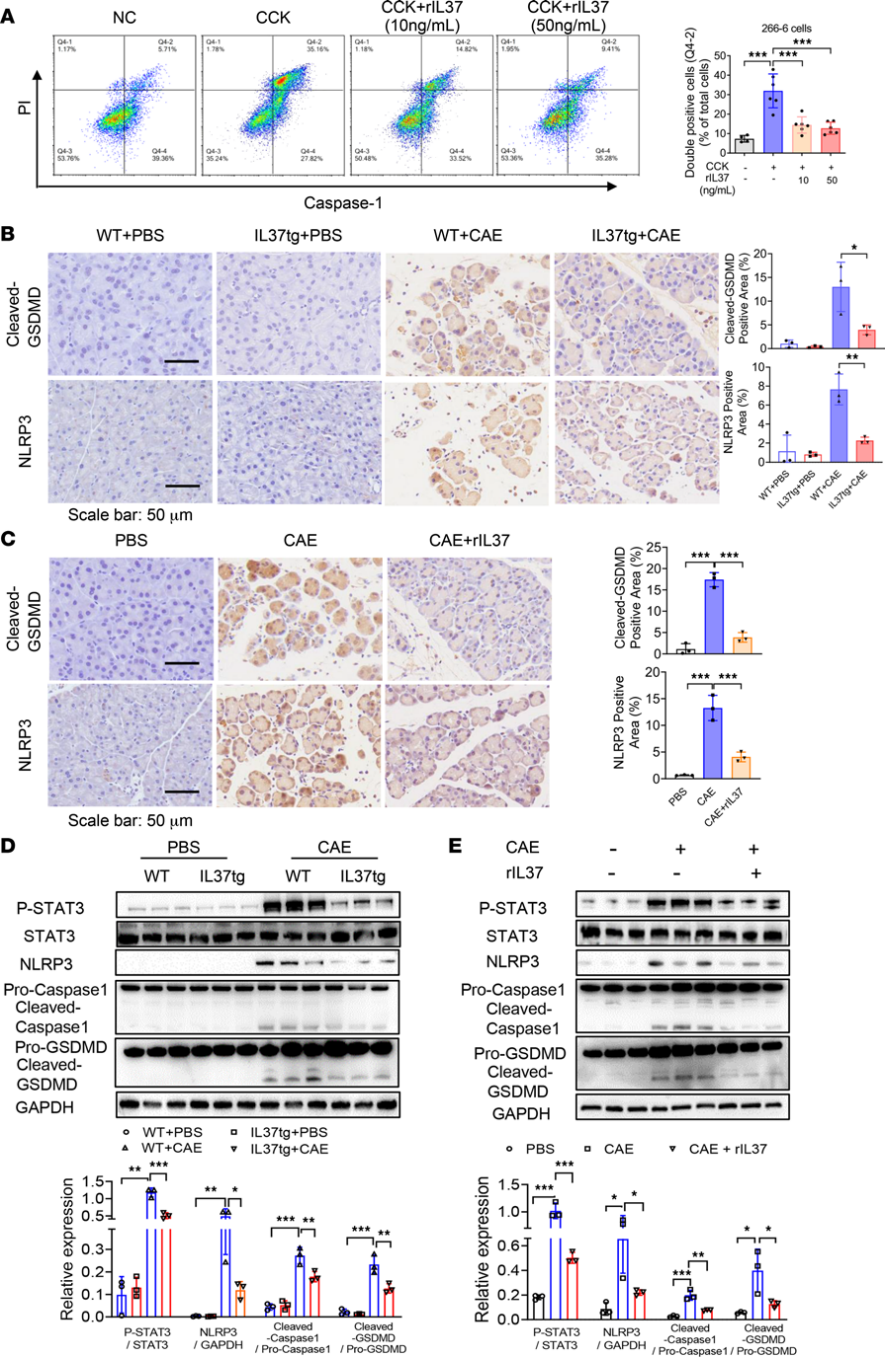
IL-37 suppresses GSDMD-mediated pyroptosis of acinar cells in AP. (**A**) 266-6 cells were stimulated with CCK and treated or not treated with rIL37 (10 or 50 ng/mL) for 12 hours. Caspase-1 and PI staining was performed to identify pyroptotic cells. Representative flow cytometry gating and proportion of double-positive cells are shown. (**B** and **C**) Pancreatic tissues collected from mice were stained with anti-GSDMD and anti-NLRP3 antibodies. (**B**) Representative images and quantitative analysis for GSDMD and NLRP3 staining of pancreatic tissues from IL37tg and WT mice with or without CAE-AP (*n* = 3 per group). (**C**) Representative images and quantitative analysis for cleaved GSDMD and NLRP3 staining in pancreatic tissues of the AP group and the rIL37-treated group (5 μg/kg) (*n* = 3 per group). Scale bars: 50 μm. (**D** and **E**) Western blot analyses and quantification of the expression of GSDMD (pro- and cleaved), NLRP3, and caspase-1 (pro- and cleaved) in pancreatic tissues from the indicated mice (*n* = 3 per group). Experiments were repeated 3 times. Statistical comparisons were made using 1-way ANOVA. Data are presented as mean ± SD, and statistical significance is denoted as *P < 0.05, **P < 0.01, and ***P < 0.001.

Previous studies reported that IL-37 blunted activation of NOD-like receptor thermal protein domain–associated protein 3 (NLRP3) inflammasome in inflammatory diseases (34, 35). Moreover, our previous studies have illustrated that the classical pathway, NLRP3 inflammasome– and GSDMD-mediated pyroptosis, plays a vital role in the progression of AP and acts as a promising therapeutic target (8, 10). Accordingly, we tested whether IL-37 affected the pyroptosis of acinar cells. Double-positive staining of caspase-1/propidium iodide (PI) in flow cytometry was recognized as detecting the presence of pyroptosis in in vitro experiments (8), and we found that the proportion of pyroptotic pancreatic acinar cells increased significantly after CCK stimulation, while IL-37 lowered the proportion of pyroptotic cells (Figure 5A). Moreover, we performed Western blotting and histochemistry analysis; expression of pyroptosis-related proteins was upregulated in the pancreatitis condition and significantly downregulated by IL-37 (Figure 5, B–E). We also examined signal transducer and activator of transcription 3 (STAT3). STAT3 is reported as a key link between IL-37 and the pyroptosis pathway (30, 36, 37). Moreover, STAT3 plays a key role during acinar cell death (38). The results showed that phosphorylated STAT3 increased in the pancreatitis condition and that this increase was rectified by IL-37 (Figure 5, D and E).

Several forms of pancreatic acinar cell death in experimental AP have been reported in previous studies, such as autophagy (39) and apoptosis (40). We performed Western blotting analyses on key proteins of the apoptosis and autophagy pathways. Autophagy pathway–related Beclin 1 and LC3B II were elevated in pancreata when treated with CAE (Supplemental Figure 7). However, we did not observe significant downregulation of the autophagy pathway when IL-37 was administered. In addition, the apoptosis pathway was activated in AP (Supplemental Figure 7); there were no obvious changes when IL-37 was administered. Overall, these results excluded a possible role of the autophagy and apoptosis pathways in protective effects of IL-37 during experimental AP.

**Figure 6:**
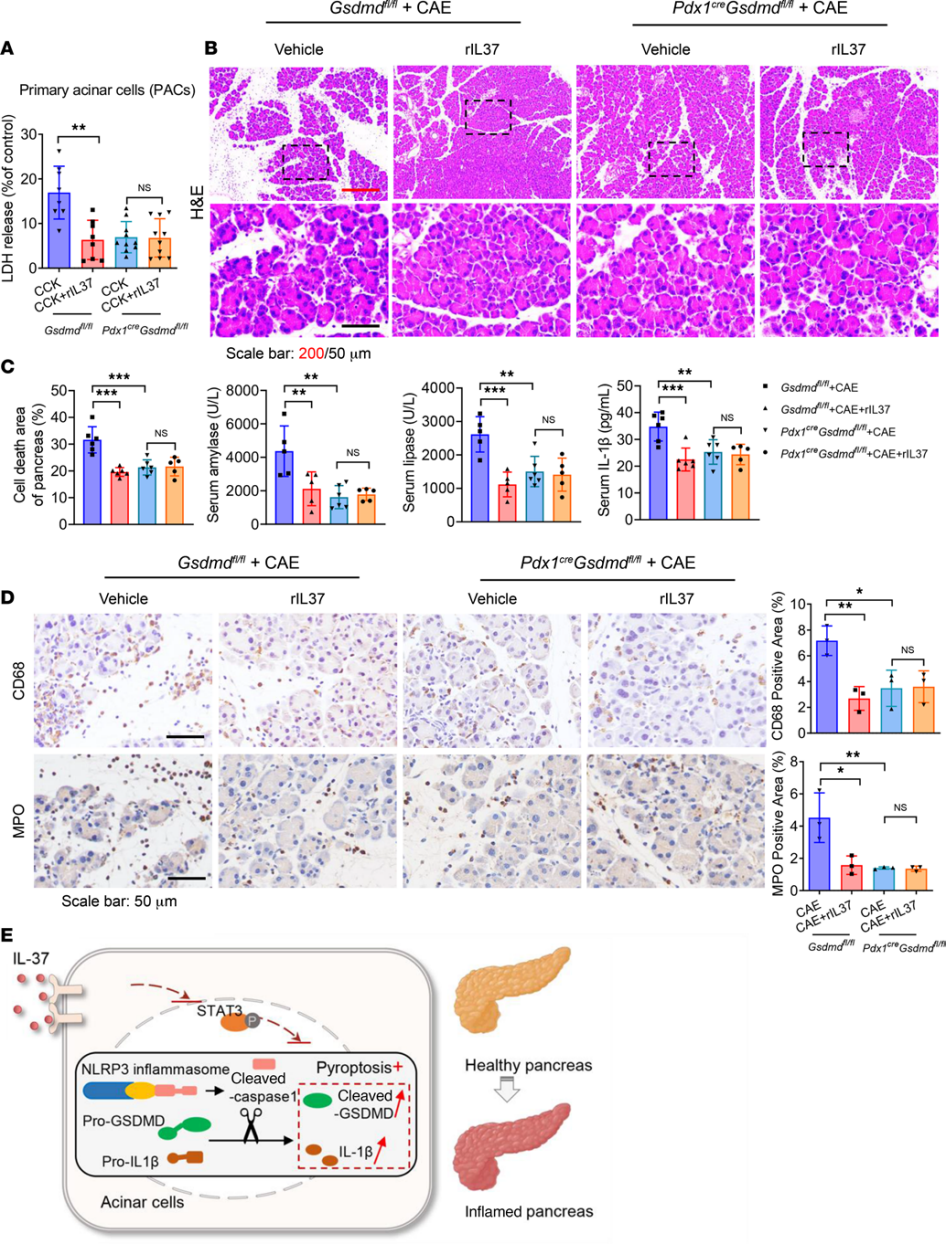
Specific knockdown of GSDMD in pancreatic acinar cells neutralizes the protective effect of IL-37. (**A**) Primary pancreatic acinar cells (PACs) were isolated from *Gsdmd^fl/fl^* and *Pdx1^cre^* *Gsdmd^fl/fl^* (G*sdmd^ΔPan^*) mice. PACs were treated with CCK with or without rIL37 (50 ng/mL). The LDH levels are shown. (**B**–**D**) AP was induced in *Gsdmd^fl/fl^* and *Pdx1^cre^* *Gsdmd^fl/fl^* mice with CAE, and rIL37 (5 μg/kg) was administered 1 hour after AP induction. (**B**) H&E staining of pancreatic tissues. Scale bars: 200 or 50 μm. (**C**) Percentages of pancreatic cell death area, and serum amylase, lipase, and IL-1β levels at 12 hours (*n* = 5–6 per group). (**D**) Representative images and quantitative analysis for IHC staining of CD68 and MPO (*n* = 3 per group). Scale bars: 50 μm. (**E**) Schematic diagram showing that IL-37 protects against AP through suppression of the pyroptosis pathway. Statistical comparisons were made using 1-way ANOVA. Data are presented as mean ± SD, and statistical significance is denoted as *P < 0.05, **P < 0.01, and ***P < 0.001.

To further verify our hypothesis, we engineered pancreas-specific GSDMD-knockout mice. Primary pancreatic acinar cells (PACs) derived from *Gsdmd^fl/fl^* and *Pdx1^cre^ Gsdmd^fl/fl^* mice were stimulated with CCK and incubated with rIL37 (using the optimal dose of 50 ng/mL validated in Figure 4). As shown in Figure 6A, administration of rIL37 did not alleviate CCK-induced cell injury in *Gsdmd*-deficient acinar cells. Furthermore, *Pdx1^cre^ Gsdmd^fl/fl^* mice were induced with the CAE-AP model, with or without rIL37 treatment (the optimal dose of 5 μg/kg validated in Figure 4). Loss of GSDMD protected against AP; however, supplementation with rIL37 did not alleviate pancreatic injury in the *Pdx1^cre^ Gsdmd^fl/fl^* mice (Figure 6, B and C, and Supplemental Figure 8). Moreover, immunohistochemical staining of macrophages and neutrophils further verified the above findings (Figure 6D). Furthermore, we used a GSDMD inhibitor, disulfiram (41), and found it exerted a significant pharmacological effect of suppressing acinar cell death and offset the effect of rIL37 on AP (Supplemental Figure 5). Collectively, IL-37 protected against AP mainly by targeting pancreatic acinar cells and by inhibiting NLRP3 inflammasome–driven and GSDMD-mediated pyroptosis (Figure 6E).

## Discussion

Though therapeutic strategies have been extensively explored, AP is still a life-threatening disease that is challenging to treat. Pancreatic necrosis (PN) is a key factor that determines the prognosis of patients with AP, causing significant mortality and reduced quality of life and requiring more invasive interventions (35, 36). Thus, prevention and treatment of PN remain a major challenge. We have confirmed the inhibitory effect of IL-37 on experimental AP and the underlying mechanism. IL-37 is a natural modulator of the immune system. Here, we explored the crucial role and potential mechanism of IL-37 in AP and found that (a) clinical data demonstrated that serum IL-37 was negatively correlated with complications such as PN; (b) IL-37 protected against acinar cell death both in vivo and in in vitro experimental AP; and (c) IL-37 rescued experimental acinar cell death dependent on the pyroptosis pathway.

It remains difficult to assess PN in clinical practice, and enhanced CT is one of the most effective auxiliary examination tools. However, it was raised in the international clinical management guideline that the timing for the initial CT assessment was at least 72–96 hours after AP onset (42), which may adversely affect the early prevention and diagnosis of PN. Here, we showed that IL-37 served as a potential biomarker of PN, considering that serum IL-37 levels were negatively correlated with disease severity, and decreased serum IL-37 was significantly associated with the occurrence of PN. According to our study, AP patients with decreased serum IL-37 may need timely CT assessment. Moreover, IL-37, as a serological index, is simple, convenient, and fast to evaluate; thus early analysis of serum IL-37 was more conducive to optimizing the diagnostic and treatment strategies for PN patients.

IL-37 has a definite antiinflammatory effect. Previous studies have reported that IL-37 elicited a unique parabolic response under multiple conditions (43). We treated mice or acinar cells with different doses of IL-37 and injected them with optimal doses of rIL37 at different times after experimental in vivo AP. There appears to be a biphasic response to dosing of IL-37 in CAE-induced pancreatitis. We supposed it to be related to the crystal structure of in vivo IL-37. A few studies illustrated that the head-to-head homodimer form of IL-37 was less effective than the monomer (44), and IL-37 existed in a monomer/dimer equilibrium, while exorbitant IL-37 concentrations would lead to dimerization and result in a dampening of its antiinflammatory activities (43). These results suggested that rIL37 rescued AP without the need for larger dosing. Thus, it should be considered for possible therapeutic interventions that maintain the stability of IL-37 at proper concentrations.

A few forms of pancreatic acinar cell death have been reported (7, 40). Our previous study used multiple genetically modified mice and showed the critical role of GSDMD-mediated pyroptosis in experimental AP cell death (8). We found that IL-37 downregulated pyroptosis-related proteins and inhibited activation of the pyroptosis executive protein GSDMD in experimental AP. Furthermore, STAT3 acts as the key transcription factor upstream of the pyroptosis pathway (45) and was involved in the pathological process of acinar cell death (38, 46). Here, we conducted a series of experiments and found that IL-37 inhibited molecules related to the pyroptosis signaling pathway. Moreover, these results were confirmed by the application of pancreatic cell–specific knockout of the key pyroptosis protein GSDMD in mice and the GSDMD inhibitor. Overall, our study adequately validated the protective effect of IL-37 on acinar cell death in experimental AP. Currently, attention is being paid to the development and application of pyroptosis inhibitors. Human-derived IL-37 may be a good choice.

Consistent with previous reports (18, 19, 21, 47), our results further verify antiinflammatory properties of IL-37 and suggest the clinical translational potential of IL-37. Regarding the safety of IL-37, it is a natural immunomodulator and exists as a precursor under physiological conditions (13, 29). Multiple studies on clinical translational practice have been conducted with recombinant proteins (48, 49). However, it remains a problem to improve purification and to lower production costs (50). One important future direction of clinical translational practice is to determine the optimal dose and therapeutic time window (TTW) of IL-37. Early supplementation with IL-37 is necessary for the following reasons: (a) Inflammation itself can activate IL-37; however, excessive activation of IL-37 results in the formation of the head-to-head homodimers and self-inhibition (12, 34, 43). (b) IL-37 protects against AP mainly by protecting acinar cells. Abnormal activation of trypsin, self-digestion of pancreatic cells, and PN are key events in the early stage of AP (7), and are also vital targets for early containment of disease progression, improvement of prognosis, and reduction of mortality (51, 52). Consequently, the determination of TTW remains a topic of general interest in pharmaceutical research. Previous studies have proposed (8) that pyroptosis inhibitors should be administered as early as possible. As our data show, IL-37 protects against AP by disrupting pyroptosis, and early administration should be considered.

In view of our findings, it remains a challenge to uncover the role of IL-37 in AP apart from protecting against PN. A comprehensive investigation is needed for future study. Our study has the following limitations. First, because our center was a clinical referral center and many of the hospitalized patients were not in the acute phase, the number of enrolled patients was limited. Therefore, multicenter studies with more samples are necessary in the future. Second, we validated the protective effect of recombinant IL-37 on experimental AP. However, the exact form of IL-37 remains unclear. Third, it was difficult to define the sole source of IL-37 in IL37tg mice, since IL-37 was generally expressed in acinar cells and non-acinar cells. Our study showed that infiltrated leukocytes were attracted to local inflammatory sites and promoted the delivery of IL-37 simultaneously. Besides, our results illustrated that BM-derived IL-37 was sufficient to exert a protective effect. However, owing to the insertion of IL-37 transgene at random, the exact originating cell component of BM was unclear. Studies concentrating on non-transgenic and non-manipulated expressed IL-37 remain to be conducted. Finally, the receptor of IL-37 on acinar cells should be uncovered in our future study.

In conclusion, this original research demonstrates that IL-37 is a promising biomarker of AP and uncovers the therapeutic potential and putative mechanism of its protection against AP. IL-37, a natural inhibitor of pancreatic acinar cell pyroptosis, has potential clinical translational prospects in future AP treatment.

## Methods

### Participants and human-derived sample collection

We recruited patients in the acute phase of AP who had been admitted to the Center of Severe Acute Pancreatitis, Department of Critical Care Medicine of Jinling Hospital, between February 2018 and December 2019. AP was diagnosed according to the International Atlanta Symposium on Acute Pancreatitis (25). Demographic, clinical, and laboratory parameters of 94 cases were included. The exclusion criteria were as follows: (a) individuals younger than 18 or older than 75 years of age; (b) pregnant women; (c) individuals diagnosed with malignant tumor; (d) individuals with chronic or recurrent pancreatitis; (e) individuals without systemic laboratory evaluation. We collected serum samples at the first day of admission from acute-phase patients. Moreover, 39 healthy volunteers were recruited as healthy controls. This study was approved by the Institutional Ethics Committee of Nanjing Jinling Hospital, affiliated with the Medical School of Nanjing University.

### ELISA

Concentrations of human serum IL-37 were detected with a Human IL-37/IL-1F7 ELISA Kit (DY1975-05, R&D Systems) following the manufacturer’s instructions. Mouse serum IL-1β levels were measured using IL-1β ELISA kits (88-7013, Thermo Fisher Scientific).

### Animals and ethics

Male C57BL/6 mice (6–8 weeks old, 20–25 g weight) and male ICR mice (6–8 weeks old, 25–30 g weight) were purchased from the Model Animal Research Center of Nanjing University (Nanjing, China), where all animals were housed under specific pathogen–free (SPF) conditions. The IL37tg mice on a C57BL/6 background were from Cyagen Biosciences Inc. Briefly, human IL-37 cDNA (NM_014439.3) was cloned and then inserted downstream of the CMV promoter in pRP [Exp] vector (VB150824-10008) (20, 24). Besides, *Pdx1^cre^* mice (International Mouse Strain Resource [IMSR] catalog JAX:014647, RRID: IMSR_JAX:014647, The Jackson Laboratory) and *Gsdmd^fl/fl^* (Model Animal Research Center of Nanjing University, Nanjing, China) were intercrossed to generate pancreatic GSDMD-specific knockout (*Pdx1^cre^ Gsdmd^fl/fl^*, *Gsdmd*^ΔPan^) (8).

All animal experiments were conducted in accordance with the Principles of Laboratory Animal Care, and were approved by the Experimental Animal Ethics Committee of Jinling Hospital, affiliated with Nanjing University.

### Animal models and treatments

#### Caerulein-induced AP.

After overnight fasting, mice were randomly divided into control and model groups. The model group was established by i.p. injection with CAE (50 or 200 μg/kg, 1-hour interval, 10 times total; NJPeptide), while the control group received equal PBS. A dose of 200 μg/kg was chosen in our in vivo experiments for better observation of PN (8, 53). Recombinant human IL-37–treated (1975-IL-025, R&D Systems) groups were set according to the specific experimentation, and rIL37 supplements were administered at varying times and doses. We collected blood samples 12 hours after the first injection and sacrificed mice 12 or 24 hours later.

#### TLCS-induced AP.

First, a retrograde injection of 2.5% TLCS (2 mg/kg; Sigma-Aldrich) was administered to the pancreatic bile duct of each mouse after anesthesia with 5% chloral hydrate for 10 minutes. The sham group that did not receive a retrograde injection was set as a control group. Additionally, to prevent excessive dehydration, normal saline was dripped into each mouse’s open abdomen. Finally, all ligatures were removed, and the abdomen was layered off. Blood samples were collected from the inner canthus at 12 hours after administration, and all animals were sacrificed 24 hours later (54).

#### ARG-induced AP.

WT and IL37tg mice were randomly separated into control (equal PBS) and model groups. Eight percent ARG (3.3 g/kg, pH 7.4, 1-hour interval, 3 total times; Sigma-Aldrich) was injected to induce AP (55). Blood samples were collected at 24 and 48 hours after the first injection, and mouse pancreatic tissues were harvested at 72 hours after the first injection.

#### Sample collection and serological detection.

All animals were sacrificed after anesthesia with 5% chloral hydrate. Samples of the pancreatic tissue were fixed in 4% paraformaldehyde for H&E and histochemical staining. The remaining pancreatic tissues were frozen at –80°C for later use. Blood samples were centrifuged, and the supernatants were preserved for hematological detection. Serum amylase (BioTechnology & Science Inc.), lipase (Nanjing Jiancheng Corp.), and IL-1β levels were measured.

### Treatment with the GSDMD inhibitor disulfiram

Disulfiram (DSF) was used as an effective GSDMD inhibitor (41). C57BL/6 WT mice were treated with DSF (50 mg/kg, i.p.). DSF was injected at 24 hours and 18 hours before CAE injection, and was injected again 2 hours after the first CAE injection according to the procedure published previously (56).

### Bone marrow transplantation

Based on previous studies (57), a BM transplantation experiment was conducted to explore the function of BM-derived IL-37. C57 mice (male, 8 weeks old, 20–25 g) were irradiated with x-rays at a dose of 9.5 Gy. In addition, BM of IL37tg and WT mice was extracted, and cells were isolated and counted. Each irradiated WT mouse was injected i.v. with 5 × 10^6^ resuspended cells, then fed antibiotic water and kept under SPF conditions for 8 weeks of recovery before AP induction.

### Pancreas histological and immunohistochemistry analyses

The paraffin-embedded pancreatic tissues were sliced for H&E and immunohistochemical staining. Briefly, we detected the expression of MPO, CD68, GSDMD, and NLRP3 in pancreatic tissues using immunohistochemical staining. Paraffin sections were dewaxed and rehydrated, then deposited and stored at 4°C overnight, then incubated with primary antibodies against MPO (1:50 dilution; ab9535, Abcam), CD68 (1:500 dilution; GB11067, Wuhan Servicebio Technology), cleaved GSDMD (1:200 dilution; sc-393581, Santa Cruz Biotechnology), NLRP3 (1:200 dilution; ab214185), and IL-37 (1:400 dilution; 60296-1-Ig, Proteintech). The next day, the corresponding secondary antibodies were applied at 20°C–25°C for approximately 50 minutes, after which prepared DAB substrate and hematoxylin were applied. The results were evaluated by 2 qualified pathologists. Images were captured using the Motic Images Plus version 2.0 system (Motic China Group Co.), and quantitative analysis of digital images was performed using ImageJ software (NIH).

### Cell lines, culture, and CCK treatment

The cancer-derived murine pancreatic acinar cell line 266-6 was purchased from the American Type Culture Collection (catalog CRL-2151, RRID: CVCL_3481). The 266-6 cells were cultured in a humidified incubator with 5% CO_2_, and were cultured in conventional medium (DMEM) containing 10% FBS (Gibco) and 1% penicillin-streptomycin (Gibco).

For in vitro studies, 266-6 cells were seeded onto a 96-well plate, then stimulated with cholecystokinin (CCK; 5–10 μM; Sigma-Aldrich) for 12 hours, either with or without rIL37 (10, 50, 250 ng/mL) at the same time (8).

### Isolation and treatment of PACs

Mouse PACs were digested and extracted by collagenase I (Sigma-Aldrich), then cultured in HEPES (Sigma-Aldrich) buffer in a 37°C incubator (58). We stimulated PACs for 6 hours with CCK, together with or without rIL37 (varying from 10 to 250 ng/mL). In addition, we extracted PACs derived from *Pdx1^cre^* *Gsdmd^fl/fl^* mice and induced an in vitro acinar injury model with CCK. Finally, cells and supernatant were collected for further investigation.

### LDH release assay

LDH release kits (LDH Cytotoxicity Assay Kits, Beyotime Biotechnology) were used to evaluate acinar cell injury according to the manufacturer’s protocols.

### Flow cytometry analysis

#### Flow cytometry of cell death.

266-6 cells were seeded in 24-well plates at a density of 2 × 10^5^ cells per well (0.5 mL/well). Then the cells were stimulated with CCK for 12 hours, with or without rIL37, at the same time. Cell pyroptosis was assessed by flow cytometry with Pyroptosis/Caspase-1 Assay Green kits (9145, ImmunoChemistry), performed according to the manufacturer’s instructions. The proportion of pyroptotic cells is presented as the percentage of double-positive cells (PI^+^/caspase-1^+^ or PI^–^PE^+^/caspase-1^–^FITC^+^) (8).

Propidium iodide (PI; 1 μmol/L; BD Biosciences) was used to detect necrotic 266-6 cells. Briefly, 266-6 cells were incubated with PI in the dark for 6 minutes. After washing with PBS, the cells were resuspended in 400 μL PBS. The CytExpert system was used for detection, and CytExpert for DxFLEX software (Beckman Inc., Shanghai, China) was used for analysis.

#### Flow cytometry detection of inflammatory cells.

As performed previously (59), the primary pancreatic leukocytes were isolated using the collagenase IV (Sigma-Aldrich) digestion method. The obtained single-cell suspension was stained with the following antibodies: PE/Cy7-CD4 (clone RM4-5), PerCP/Cy5.5-CD11b (clone M1/70), APC/Cy7-CD11c (clone N418), BV421-F4/80 (clone BM8), and AF488-CD206 (clone C068C2), all purchased from BioLegend. APC–TNF-α antibody was from Thermo Fisher Scientific (17-7321-81). Macrophages were then circle-gated and their activity was evaluated. Briefly, CD11b^+^F4/80^+^TNF-α^+^ cells represented M1 macrophages and CD11b^+^F4/80^+^CD206^+^ cells represented M2 macrophages. The final data were analyzed with FlowJo version 10.0 (Stanford University, Stanford, California, USA).

Data are presented as dot plots, histograms, and pseudo-color images, and the results of statistical analysis are presented as the percentage of total target-gated events (at least 8,000 events per sample).

### Protein extraction and Western blot analysis

Briefly, pancreatic tissues were lysed in RIPA lysis buffer, and total proteins were extracted. Concentrations of proteins were measured using the BCA protein assay kit (Pierce, Thermo Fisher Scientific) following the commercial instructions. Equivalent protein (approximately 30 μg/sample) was separated by SDS-PAGE gel and then transferred to PVDF membranes. Then the membranes were blocked for 1 hour in 5% nonfat milk. Afterward, membranes were incubated with primary antibodies against GAPDH (1:1,000 dilution; Sigma-Aldrich), NLRP3 (1:1,000 dilution; D4D8T, Cell Signaling Technology), GSDMD (1:500 dilution; ab219800, Abcam), caspase-1 (1:500 dilution; AG-20B-0042, AdipoGen), STAT3 (1:1,000 dilution; A19566, Abclonal), phosphorylated STAT3 (1:1,000 dilution; AP0705, Abclonal), Beclin 1 (1:1,000 dilution; A21191, Abclonal), LC3B (1:1,000 dilution; ab48394, Abcam), Bax (1:1,000 dilution; 29057, Sabbiotech), and Bcl-2 (1:1,000 dilution; ab196495, Abcam) overnight at 4°C. Finally, HRP-conjugated secondary antibodies were applied for 1 hour. The proteins were detected and analyzed by an ECL Plus chemiluminescence imaging system (Tanon).

### Quantitative real-time PCR

Briefly, quantitative real-time PCR was conducted to analyze the transcription of mRNA as described in previous studies (59). Total RNA of pancreatic tissue from different treatment groups was isolated with TRIzol reagent. Then reverse transcription of RNA was performed following the commercial kits’ instructions. Additionally, GAPDH was used as endogenous control. The primers involved were as follows: mGAPDH forward, 5′-AGGTCGGTGTGAACGGAT-TTG-3′; mGAPDH reverse, 5′-TGTAGACCATGTAGTTGA-GGTCA-3′; IL-37 forward, 5′-TTCTTTGCATTAGCCTCATCCTT-3′; IL-37 reverse, 5′-CGTGCTGATTCCTTTTGGGC-3′.

### Statistics

Data were analyzed by IBM SPSS 26.0 and GraphPad Prism 9.0. In addition, a heatmap of variables was performed using the R corrplot package. Normal continuous variables are presented as the mean ± SD. Non-normal continuous variables were described as median (IQR). Categorical variables were described as frequency (percentage). Normal continuous variables were compared by Student’s 2-tailed *t* test or 1-way ANOVA, whereas non-normal continuous variables were analyzed by Mann-Whitney test or Kruskal-Wallis test. Categorical data were analyzed by χ^2^ test. Receiver operating characteristic curve and area under the curve were applied to determine the optimal cutoff values of serum IL-37. Logistic regression model was established to evaluate the risk factors for poor clinical outcomes. Variables with statistical significance in univariate analysis (*P* < 0.10) were involved in multivariable logistic regression analysis. Statistical significance was defined as *P* less than 0.05 (2-tailed).

### Study approval

All animal experiments were conducted in accordance with the *Principles of Laboratory Animal Care* (NIH publication 85Y23, revised 1996), and were approved by the Experimental Animal Ethics Committee of Jinling Hospital, affiliated with Nanjing University (2018GKJDWLS-03-157). All procedures followed were in accordance with the principles of the Declaration of Helsinki, and the study was approved by the Institutional Ethics Committee of Nanjing Jinling Hospital, affiliated with the Medical School of Nanjing University.

## Author contributions

NM, CY, and JS contributed to study design, data curation, and manuscript writing. QZ and XM contributed to investigation and methodology. YL and BL contributed to clinical data analysis. WG contributed to material or technique support and critical revision of the manuscript. GL, JX, JL, and WL contributed to study design, conceptualization, and funding acquisition. All authors read and approved the manuscript.

## Supplementary Material

Supplemental data

## Version 1 09/27/2022

In-Press Preview

## Version 2 11/08/2022

Electronic publication
