# Supplementary material for: Interleukin-37 protects against acinar cell pyroptosis in acute pancreatitis
Source: JCI Insight. 2022 Nov 8;7(21):e161244. doi: 10.1172/jci.insight.161244 (PMC9675483; doi:10.1172/jci.insight.161244)
Supplement: Supplemental data [file jciinsight-7-161244-s250.pdf]

SUPPLEMENTAL FIGURE LEGENDS

Supplemental Figure 1. Bone marrow-derived IL-37 protected mice from experimental AP. (**A**) Representative images and quantitative analysis for immunohistochemically labeling of IL-37 in pancreata (n = 4 per group), scale bar= 50 μm. (**B**) Schematic diagram of bone marrow transplantation from IL37tg and WT littermates to C57 WT recipients. AP was induced with CAE under SPF conditions for eight weeks (n = 6 per group). (**C**) H&E staining of pancreata at 24 hours. (**D**) Percentages of pancreatic cell death area. Serum amylase and lipase levels at 12 hours. Statistical comparisons were made using student t test or one-way ANOVA. Data are presented as the mean ± SD, and statistical significance is denoted as $^{**}P < 0.01$ and $^{***}P < 0.001$.

Supplemental Figure 2. The protective effect of IL37 was observed when mice were treated with caerulein at 50 μg/kg. (**A-B**) WT and IL37tg mice were injected with CAE (50 μg/kg, one-hour intervals, 10 times in total), and PBS was injected as control. (**A**) H&E staining of pancreatic tissues. (**B**) Percentages of pancreatic cell death area. Serum amylase, and lipase levels at 12 hours. (**C-D**) WT mice were injected with CAE and treated with rIL37 (5μg/kg, one hour after CAE injection). (**C**) H&E staining of pancreatic tissues. (**D**) Percentages of pancreatic cell death area. Serum amylase, and lipase levels at 12 hours. Statistical comparisons were made using one-way ANOVA. Data are presented as the mean ± SD, and statistical significance is denoted as $^{**}P < 0.01$ and $^{***}P < 0.001$.

Supplemental Figure 3. IL37 overexpression alleviated AP at 12h. (**A**) Timeframe of CAE induced AP. (**B**) H&E staining of pancreata at 12 hours. (**C**) Percentages of pancreatic cell

death area. Serum amylase and lipase levels at 12 hours. Experiments were repeated three times. Statistical comparisons were made using one-way ANOVA. Data are presented as the mean ± SD, and statistical significance is denoted as $^{***}P < 0.001$.

Supplemental Figure 4 Quantitative analysis for IHC staining of CD68 and MPO in pancreatic tissues. (**A**) Quantitative analysis for IHC staining of CD68 and MPO from IL37tg and WT mice that were treated with or without TLCS (n = 4 per group). (**B**) Quantitative analysis for IHC staining of CD68 and MPO of IL37tg and WT mice that were treated with or without ARG (n = 4 per group). Statistical comparisons were made using one-way ANOVA. Data are presented as the mean ± SD, and statistical significance is denoted as $^{***}P < 0.001$.

Supplemental Figure 5. Administration with rIL37 at different time points protected against AP and this effect could be counteracted by pyroptosis inhibitor. C57BL/6J WT mice were injected with CAE to induce AP, rIL37 were administrated one, three, and six hours after the first CAE injection (n=6 per group). Moreover, GSDMD inhibitor disulfiram (DSF) were injected at 50 mg/kg. (**A**) H&E staining of pancreata at 24 hours. (**B**) Percentages of pancreatic cell death area. Serum amylase and lipase levels at 12 hours. Statistical comparisons were made using one-way ANOVA. Data are presented as the mean ± SD, and statistical significance is denoted as $^{*}P < 0.05$, $^{**}P < 0.01$, and $^{***}P < 0.001$. No statistical significance is denoted as *ns*.

Supplemental Figure 6. Prophylactic administration with rIL37 reduced pancreatitis. The time dependency of the effect of rIL37 on AP progression, WT mice were randomly divided into PBS, CAE-induced AP model, and AP+rIL37 (5μg/kg, pre-one hour) prevention

groups. (**A**) H&E staining of pancreata at 24 hours. (**B**) Percentages of pancreatic cell death area. Serum amylase and IL-1β levels at 12 hours. (n = 5–6 per group). Statistical comparisons were made using one-way ANOVA. Data are presented as the mean ± SD, and statistical significance is denoted as $^{*}P < 0.05$, $^{**}P < 0.01$, and $^{***}P < 0.001$.

Supplemental Figure 7. IL37 rescued AP independent of autophagic or apoptotic pathway. (**A-B**) Western blot analyses and qualification of the expression of Beclin1, LC3B (II/I), Bax, and Bcl-2 in pancreatic tissues (n = 3 per group). Experiments were repeated three times. Statistical comparisons were made using one-way ANOVA. Data are presented as the mean ± SD, and statistical significance is denoted as $^{*}P < 0.05$, $^{**}P < 0.01$, and $^{***}P < 0.001$.

Supplemental Figure 8. Western blot analysis of GSDMD expression in $Gsdmd^{fl/fl}$ and $Pdx1^{cre}Gsdmd^{fl/fl}$ mice. (**A**) Western blot analyses and qualification of the expression of GSDMD (pro- and cleaved-) in pancreatic tissues from $Gsdmd^{fl/fl}$ and $Pdx1^{cre}Gsdmd^{fl/fl}$ mice treated with or without caerulein (n = 3 per group). Statistical comparisons were made using one-way ANOVA. Data are presented as the mean ± SD, and statistical significance is denoted as $^{*}P < 0.05$, $^{**}P < 0.01$, and $^{***}P < 0.001$.

Supplemental Figure 1

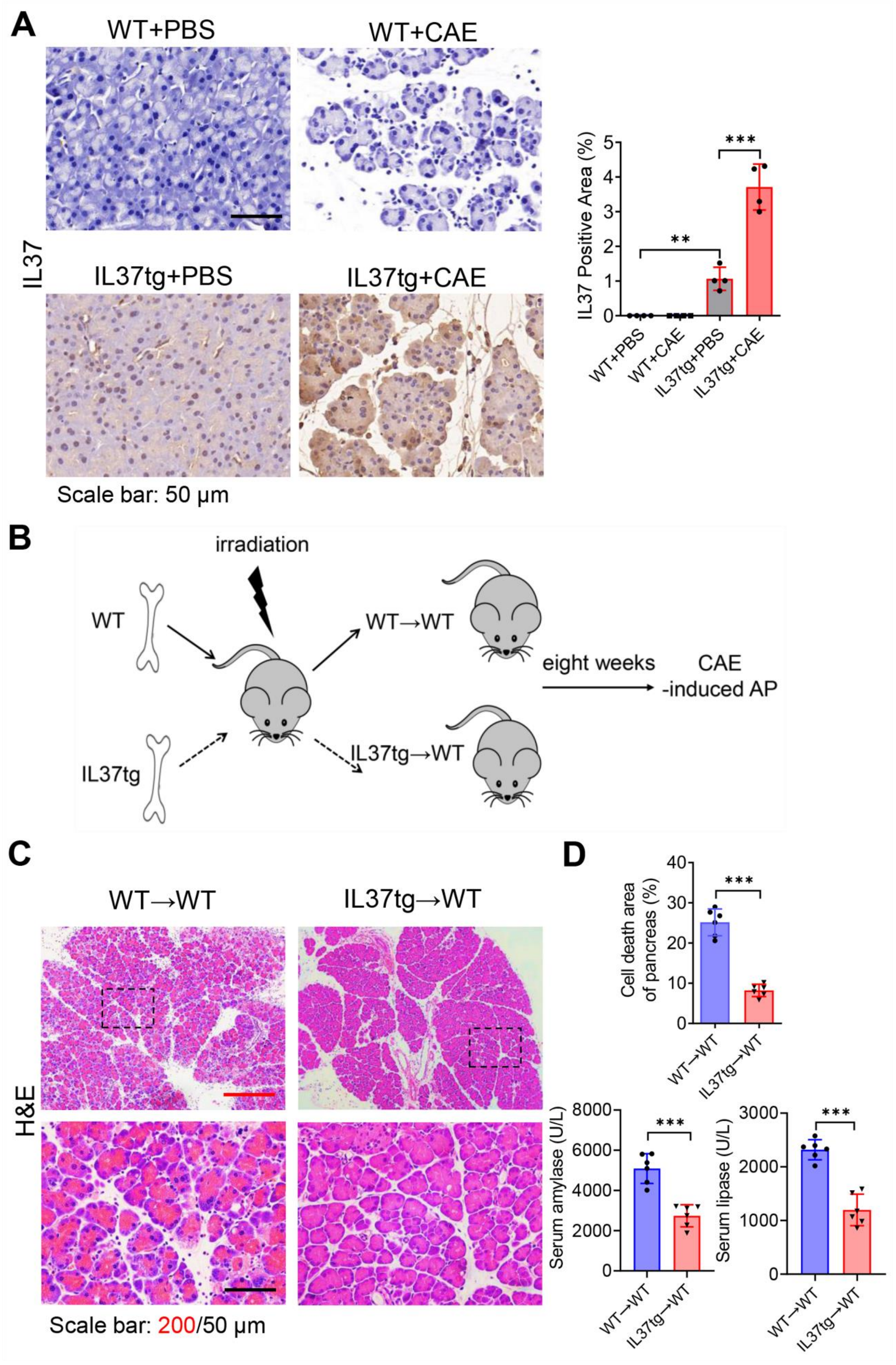

Supplemental Figure 2

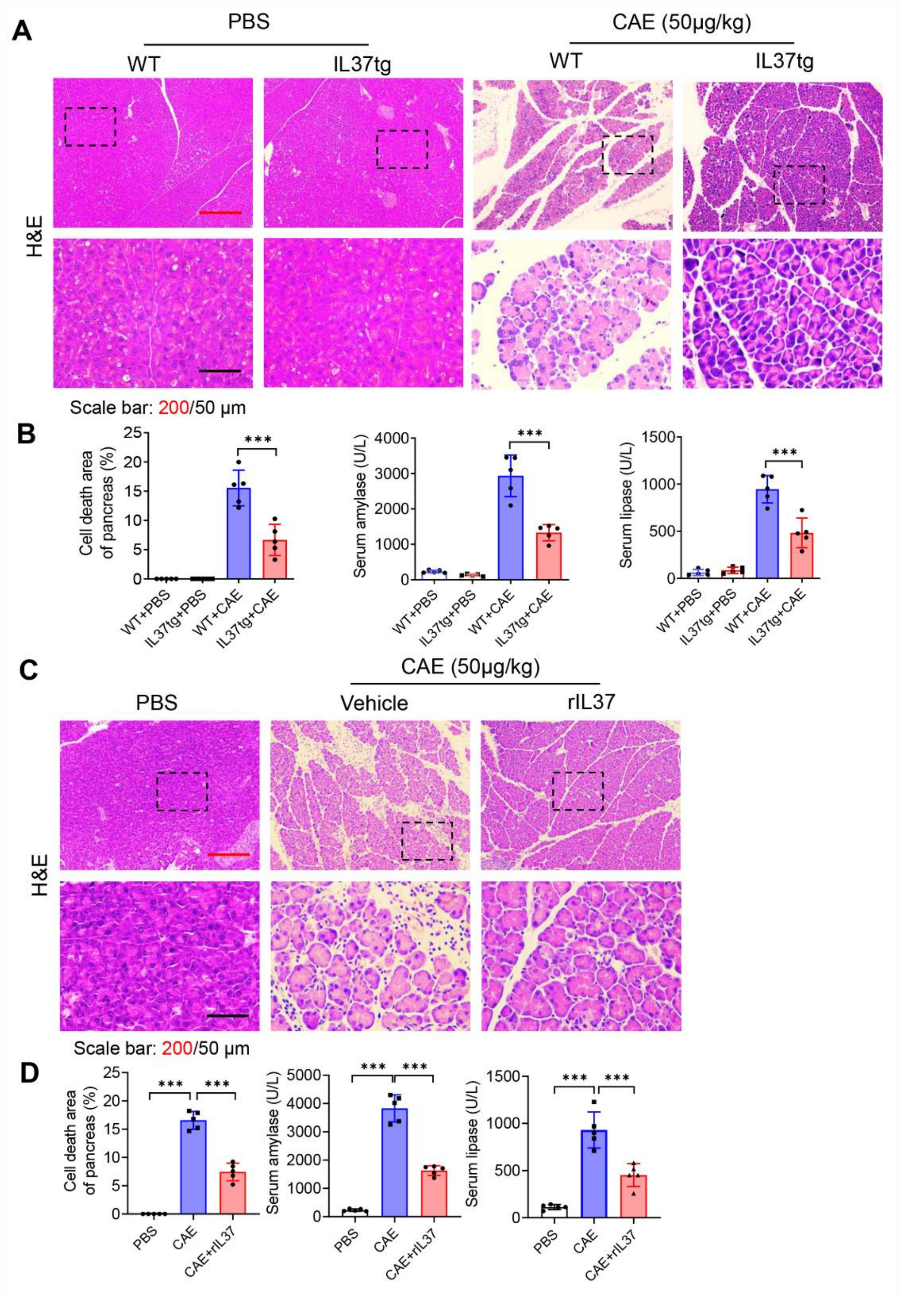

Supplemental Figure 3

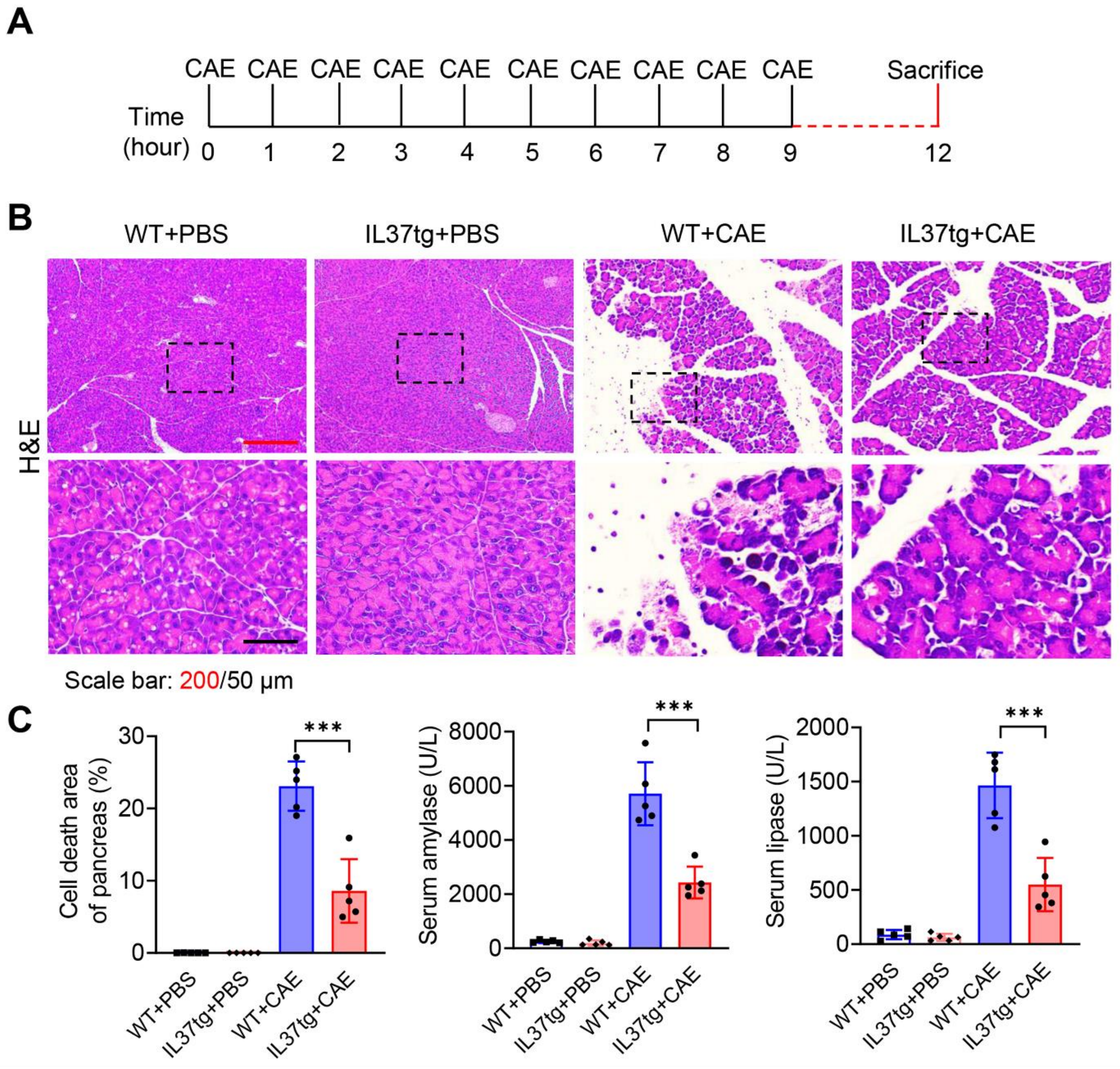

Supplemental Figure 4

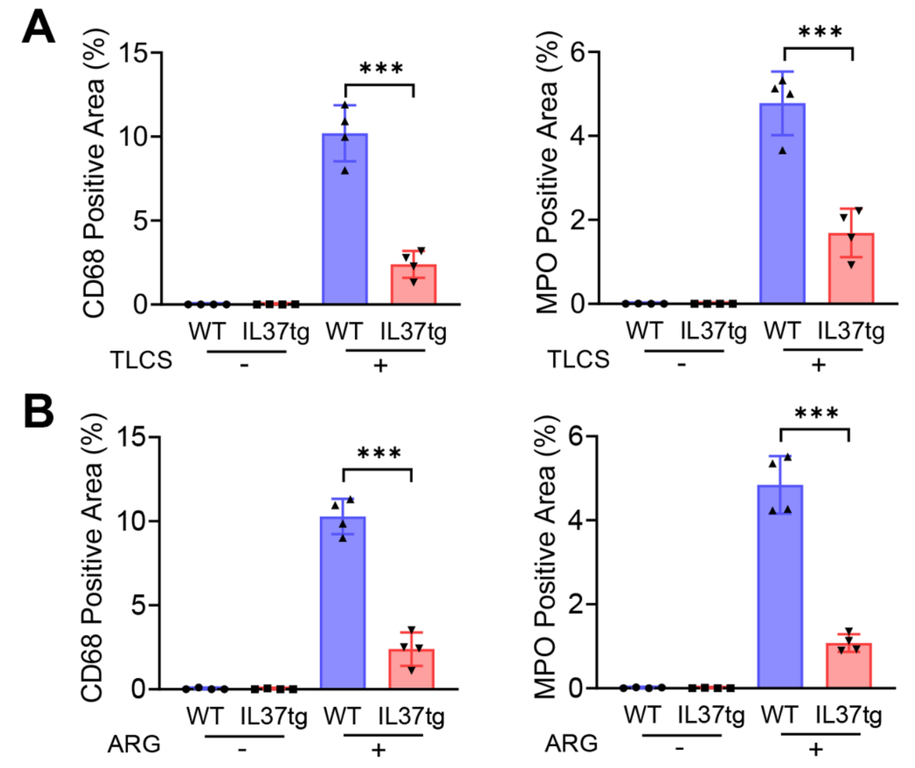

Supplemental Figure 5

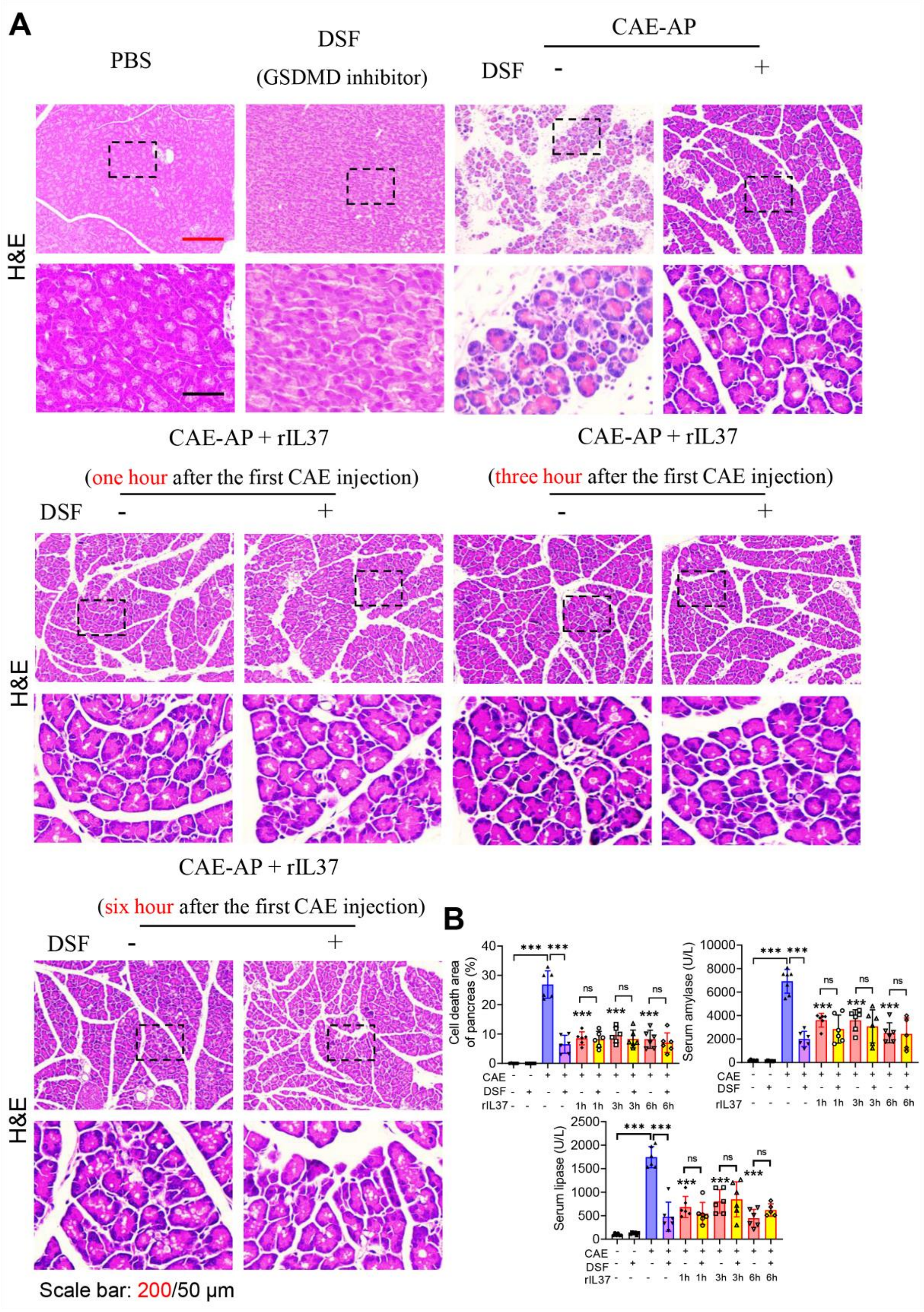

Supplemental Figure 6

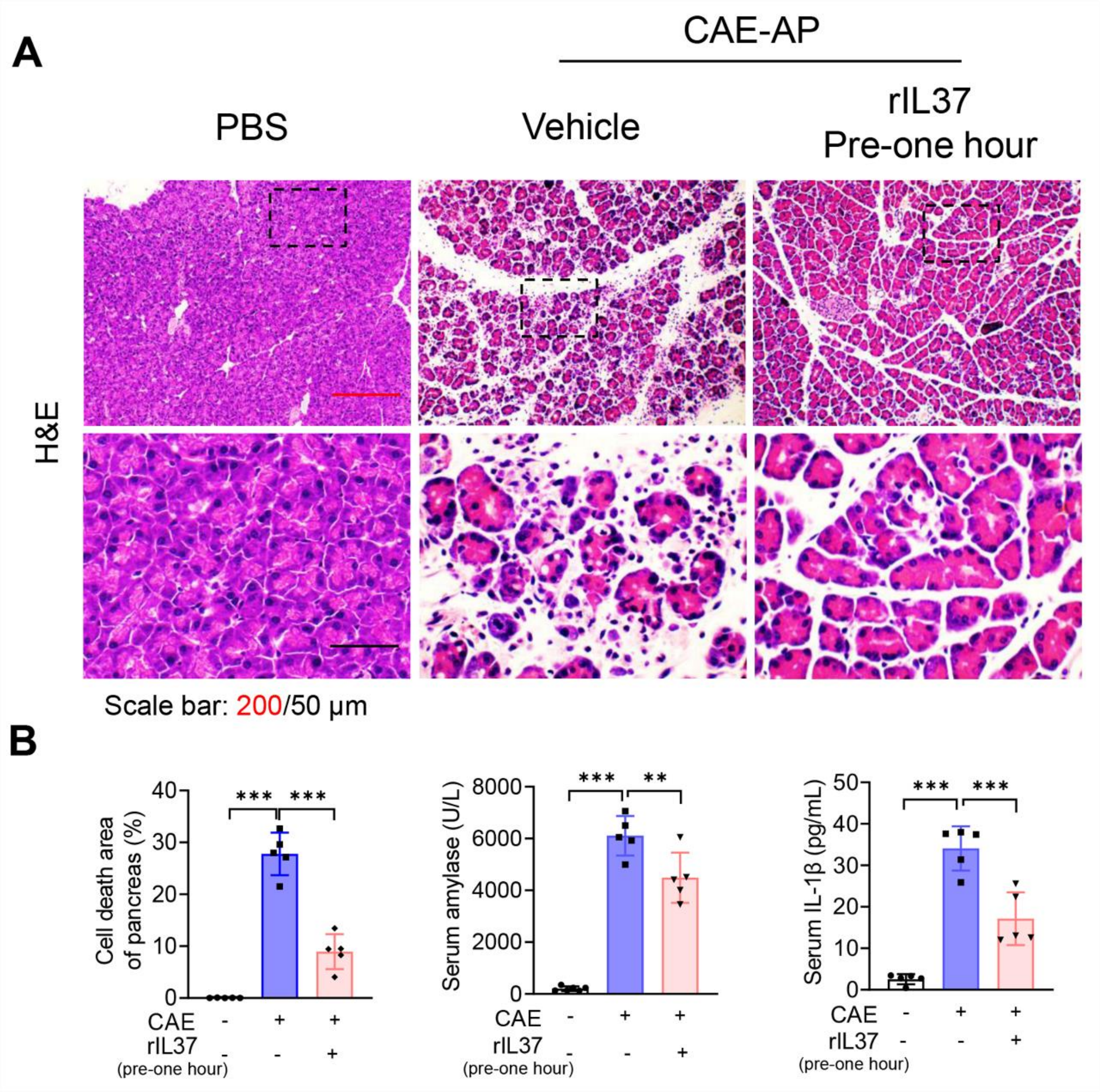

Supplemental Figure 7

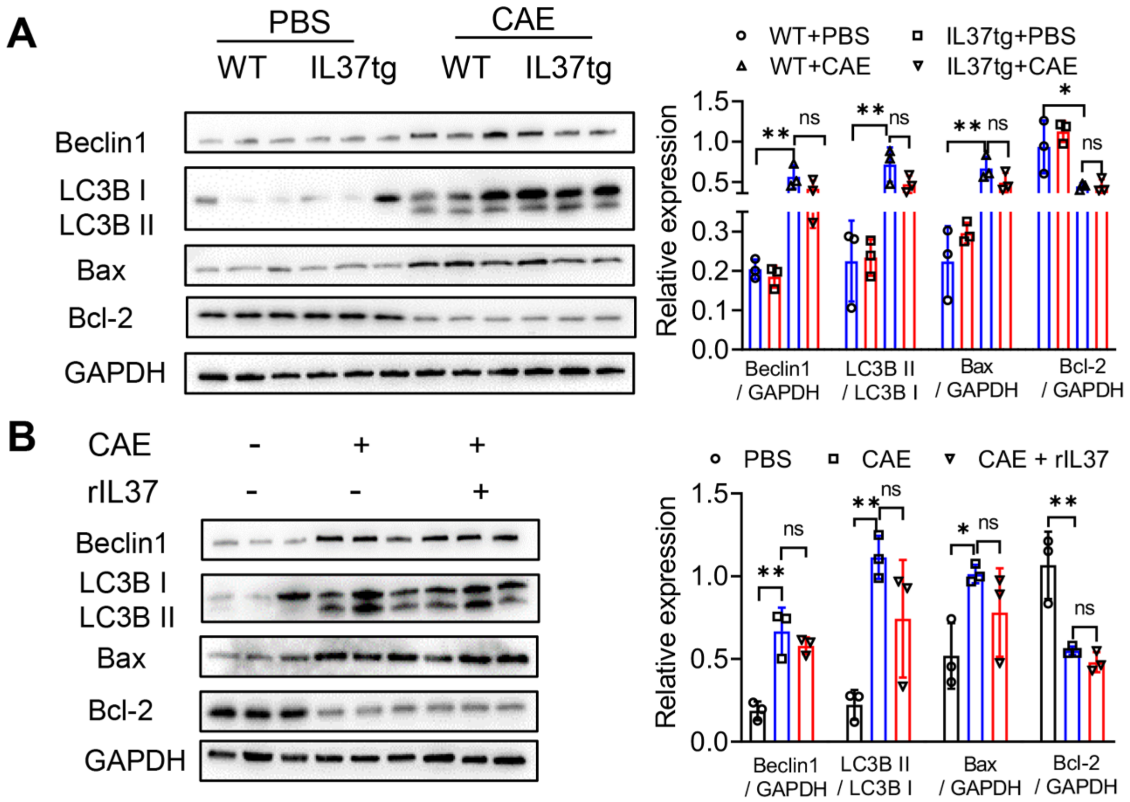

Supplemental Figure 8

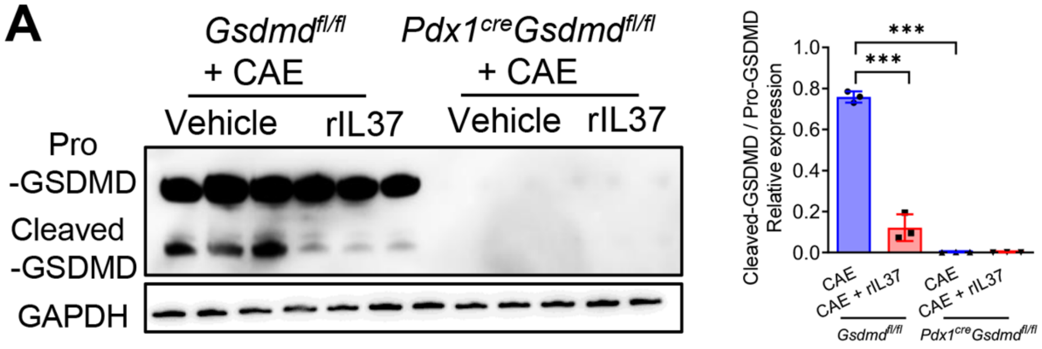

Supplemental Table 1 Univariate and multivariate analyses of risk factors for pancreatic necrosis in AP patients.

Variables	Non-PN group	PN group	Univariate analysis		Multivariable analysis	
	N=34	N=60	*P* value	OR (95% CI)	*P* value	OR (95% CI)
**Age**, years	42 ±15	42 ±12	0.942	1.00 (0.97-1.04)		
**Gender**, Male	23 (67.6%)	47 (78.3%)	0.256	0.58 (0.23-1.45)		
**BMI**, kg/m^2	25.49 ±3.77	27.44 ±3.88	0.044	1.16 (1.00-1.34)	0.734	1.04 (0.84-1.27)
**Smoking**	12 (35.3%)	17 (28.3%)	0.483	0.72 (0.29-1.78)		
**Drinking**	8 (23.5%)	21 (35.0%)	0.250	1.75 (0.68-1.56)		
**APACHE II**	4.5 (2-7)	10 (7-14)	<0.001	1.36 (1.18-1.56)	0.048	1.2 (1.00- 1.45)
**CTSI score**	3 (2-4)	7 (5.3-8)	<0.001	4.57 (2.34-8.89)		
**Laboratory indexes at admission**						
**WBC**, $\times 10^9$/L	9.48 (6.95-11.41)	10.52 (8.24-13.18)	0.074	1.12 (0.99-1.26)	0.114	1.17 (0.96-1.44)
**PLT**, $\times 10^9$/L	167.27 ±51.54	167.98 ±69.96	0.958	1.00 (0.99-1.01)		
**HCT**, %	38.40 ±5.40	33.90 ±7.30	0.004	0.90(0.83, 0.97)	0.071	0.90 (0.80-1.01)
**Amylase**, U/L	123 (61, 255)	144 (60, 266)	0.257	1.00 (0.99-1.00)		
**Lipase**, U/L	408 (206, 1017)	568 (216, 1098)	0.996	1.00 (0.99-1.00)		
**LDH**, U/L	5.84 (4.27-7.65)	17.79 (8.32-27.73)	0.002	1.1(1.03-1.17)	0.927	1.00 (0.96- 1.05)
**CRP**, mg/L	120.30 ±77.82	188.88 ±69.74	<0.001	1.01 (1.01-1.02)	0.141	1.00 (0.99-1.02)
**IL-6**, ng/L	38.84 (15.48-95.26)	157.30 (66.14-300.05)	<0.001	1.01 (1.01-1.02)	0.207	1.00 (0.99-1.01)
**PCT**, μg/L	0.25 (0.09-0.94)	1.09 (0.38-2.17)	0.171	1.06 (0.98-1.16)		
**SCr**, μmol/L	53.25 (43.38-68.78)	63.60 (52.00-91.18)	0.067	1.01 (1.00-1.03)	0.661	1.00 (0.99-1.01)
**BUN**, mmol/L	4.25 (2.80-7.65)	4.70 (3.43-7.33)	0.889	1.01 (0.90-1.13)		
**IL-37**, pg/ml	81.49 (62.53-118.55)	55.08 (51.42-58.59)	<0.001	0.92 (0.89-0.96)	0.041	0.96 (0.93-0.99)

Data are described as the mean ± SD, median (IQR), or n (%). SD, standard deviation; OR, odds ratio; CI, confidence interval; PN, pancreatic necrosis; BMI, body mass index; APACHE II, Acute Physiology and Chronic Health Evaluation II; CTSI, Computed Tomography Severity Index; CRP, C-reactive protein; PCT, procalcitonin; WBC, white blood cell; PLT, platelets; HCT, hematocrit; LDH, lactate dehydrogenase; SCr, serum creatinine; BUN, blood urea nitrogen.
